# Mammary Paget’s Disease as a Sign of Local Recurrence Two Decades Following Breast Conservation and Adjuvant Therapy for Early Stage Breast Cancer

**DOI:** 10.7759/cureus.61521

**Published:** 2024-06-02

**Authors:** Rimsha J Afzal, Mustafa M Basree, Steven P Howard, Jin Xu, Lee G Wilke, Bethany M Anderson

**Affiliations:** 1 Medical Education, University of Pikeville - Kentucky College of Osteopathic Medicine, Pikeville, USA; 2 Radiation Oncology, University of Wisconsin School of Medicine and Public Health, Madison, USA; 3 Pathology and Laboratory Medicine, University of Wisconsin School of Medicine and Public Health, Madison, USA; 4 Breast Surgery, University of Wisconsin School of Medicine and Public Health, Madison, USA

**Keywords:** delayed recurrence, breast cancer recurrence, breast cancer detection, breast cancer management, mammary paget's disease

## Abstract

Reports of mammary Paget’s disease (MPD) as a manifestation of breast cancer recurrence are rare. MPD presents a particular challenge when emerging more than two decades after a breast cancer treated with evidence-based therapy. There is a broad spectrum of non-malignant causes for dermatitis of the nipple during the initial presentation that may delay cancer work-up. This case highlights the MPD work-up and management in the context of a personal history of breast cancer. This unique clinical presentation emphasizes the importance of vigilant cancer surveillance for timely intervention, especially for a presumed cured cancer.

## Introduction

At present, there are limited reports on patients with in-breast tumor recurrence presenting with mammary Paget's disease (MPD) two decades following comprehensive treatment [1]. MPD of the nipple-areolar complex (NAC) is a rare intraepithelial malignancy of the breast, with a 90% association with underlying breast cancer [2]. MPD is characterized by the presence of Paget cells, intraepidermal cells with distinct histopathological features, that are present within the nipple epithelium [3]. There are two theories describing the etiology of MPD [4]. The epidermotropic theory states that Paget cells arise from underlying intraductal carcinoma that have migrated along the basement membrane of the nipple [5]. The intraepidermal theory explains that MPD pathogenesis is a malignant conversion of pluripotent keratinocyte stem cells or cells of apocrine gland ducts, which is supported by cytological similarity between Paget and Toker cells - epithelial cells found in the NAC of 10% of women [5]. MPD presents as eczematous patches of scaly tissue surrounding the NAC, associated with tingling and pruritis [3]. From a histopathological perspective, Paget cells are characterized by abundant pale cytoplasm with enlarged pleomorphic, hyperchromatic nuclei and usually present as either single cells or clusters of cells spreading throughout the epidermis. Immunohistochemically, Paget cells also show a very similar staining pattern to that of the underlying breast carcinoma [6]. The recurrence of breast carcinoma with initial sign of MPD of the NAC is rarely seen in the literature; it is estimated to be around 2.2% of local failures [7]. Here we describe the case of a 74-year-old patient with local recurrence of breast cancer more than 20 years following prior definitive treatment.

## Case presentation

A 74-year-old female was diagnosed with clinical T2N0M0 (stage IIA) invasive ductal carcinoma (IDC) involving the right upper inner breast quadrant in 2000 at age 51. She underwent breast-conserving surgery (BCS) and sentinel lymph node surgery, which demonstrated a 1.6-cm well-differentiated, estrogen receptor (ER) and progesterone receptor (PR) positive, HER-2 negative carcinoma with zero out of five involved lymph nodes. Peritumoral lymphatic invasion was present. She then underwent adjuvant whole breast radiation to a dose of 50.4 Gy followed by 10 Gy boost to the lumpectomy cavity. Endocrine therapy, including tamoxifen and anastrozole, was poorly tolerated with severe hot flashes and discontinued after roughly 2.5 years.

**Figure 1:**
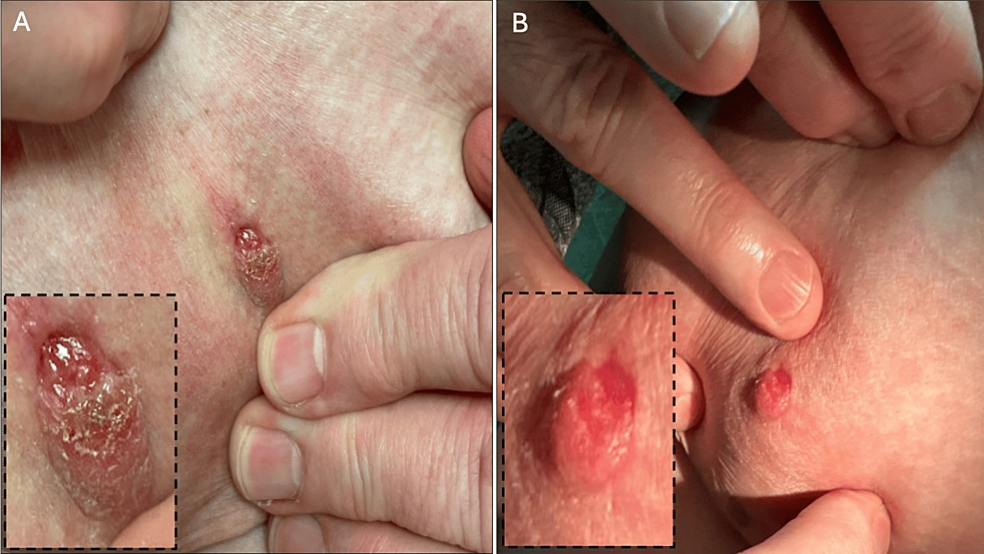
Physical examination at the time of office visit showing retraction of the nipple with a small bright red lesion with adjacent crusting (panels A and B).

**Figure 2:**
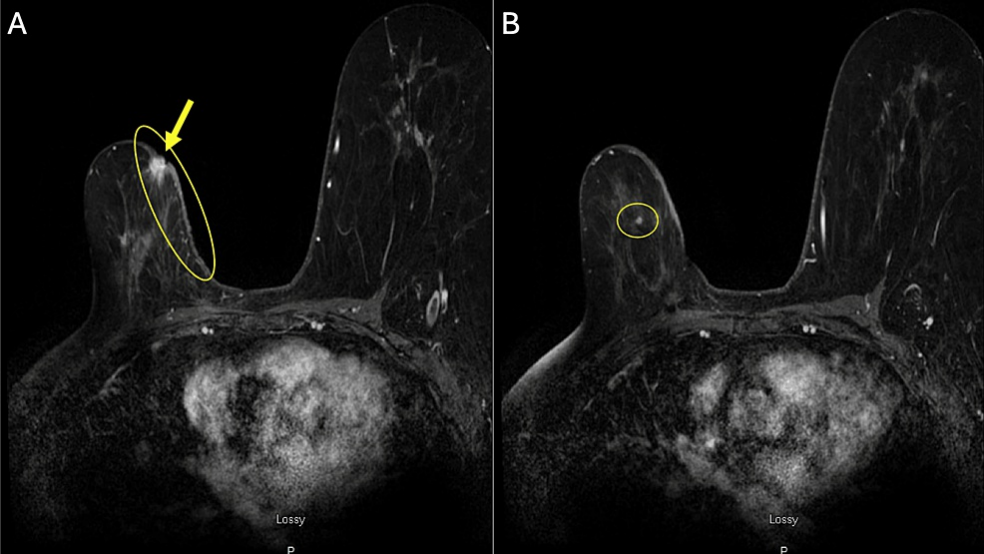
Axial T1 post-contrast MRI images of the right breast demonstrating skin thickening (panel A, ovoid) with associated enhancement of the nipple-areolar complex (panel A, arrow). Panel B shows a mass at 12 o'clock, measuring up to 5 mm.

She remained disease-free, undergoing annual mammograms, until 2022 when she presented to her primary care provider with a four-month history of waxing and waning skin irritation involving the right nipple. This was associated with dryness, mild redness, and occasional crusty discharge. A trial of antibiotics transiently improved her symptoms but did not resolve the irritation. She was seen at the radiation oncology clinic with examination demonstrating a small, roughly 1.0 cm area of erythema with eczematous and central yellow crusting of the right nipple. It was felt that it represented irritation from chronic nipple inversion since her earlier breast cancer diagnosis (Figure 1). However, given her prior history of breast cancer and persistent nature of the lesion, she underwent a punch biopsy, which revealed MPD. On immunohistochemistry (IHC), lesion was positive for CK7 and HER2 but negative for ER and PR, supporting the diagnosis of MPD. Diagnostic mammogram and ultrasound of the right breast did not demonstrate evidence of malignancy. However, breast MRI showed enhancement of the right NAC with a 0.5 cm mass at the 12 o’clock position suspicious for malignancy (Figure 2). Biopsy of the central mass demonstrated a grade 2 IDC, ER- and PR-positive without HER2 amplification with Ki67 of 5%. She underwent right simple mastectomy with sentinel lymph node biopsy (SLNB), with final pathology demonstrating stage IA disease (pT1a pN0[sn]). She elected to not undergo adjuvant endocrine therapy given the low risk of recurrence post-mastectomy, as well as prior intolerance. She remains disease-free roughly one year after her surgery.

## Discussion

This case of MPD highlights a unique clinical sign for breast cancer diagnosis in the context of previously treated ipsilateral breast cancer after roughly two decades in remission. Two published case studies highlight MPD recurrence 10 years after treatment in women who underwent breast reconstruction and nipple-sparing mastectomy (NSM) [8]. Another is a report of MPD at a prior core needle biopsy site 1.5 years after the biopsy [9]. The presentation of the disease being limited to the nipple subverts attention from early detection and confirmation of MPD [10]. Differential diagnosis for women presenting with MPD includes underlying dermatological conditions, drug or autoimmune skin reaction, primary, recurrent, or radiation-induced breast malignancy.

There are several independent factors associated with a high risk of late recurrence with MPD. Older and post-menopausal women have an increased prevalence of MPD [10]. Case reports citing MPD as a sign of recurrence years later have reported the diagnostic pattern of the initial tumor to be intermediate grade, ER+, PR+, and HER-2 non-amplified. In a retrospective study analyzing clinicopathological features between MPD and non-MPD patients with invasive breast cancer, patients with MPD showed higher proportions of proliferation index marker Ki67 [11]. Aside from having hormone-positive disease, our patient had a unique presentation with lower Ki67, no nodal involvement, and not having undergone NSM. In another case of MPD, endocrine therapy was discontinued prematurely due to intolerance [1], similar to our patient.

Diagnostic imaging, including mammography and MRI, following confirmation of MPD is important in ruling out underlying malignancy. No high-quality, prospective data exist to guide management for patients with MPD versus those with non-MPD ipsilateral breast cancer recurrence. Moreover, it is challenging to know if the new breast cancer is recurrent or new primary. One may possibly ascertain the etiology via sequencing and gene expression analysis [12,13]. Unfortunately, there was not enough tissue to complete this test in our patient. Regardless of underlying etiology, management is similar. Surgical approach for recurrent disease has historically been mastectomy for patients who have undergone BCS and adjuvant radiation in the past. BCS alone is associated with high rates of in-breast recurrence if not coupled with adjuvant radiation [14]. Emerging data provide evidence for safety and efficacy of breast conservation with adjuvant re-irradiation in select women [15,16]. Our patient underwent mastectomy instead of repeat BCS and adjuvant radiation given considerable breast shrinkage after her original treatment course. Moreover, although there is a move to omit SLNB in older patients with early stage disease [17,18] this was not pursued in our patient due to her prior ipsilateral breast cancer. Nodal evaluation in breast malignancy is similar irrespective of surgical management as historically up to 40% of women with cN0 disease harbor nodal disease [19]. A recent meta-analysis of real-world data from 38 studies on the management of MPD compared outcomes of women who underwent mastectomy, BCS alone, or BCS with radiotherapy. Results showed that local recurrence was significantly lower in women with MPD and underlying carcinoma who underwent mastectomy (5.9%) and BCS with adjuvant radiation (8%) compared to BCS alone (21.2%) [10].

## Conclusions

This case emphasizes the importance of awareness of the potential for late locoregional recurrence to prevent delays in care. This unique presentation highlights the need for vigilant cancer surveillance, even for cancers believed to be cured. New skin lesions involving the NAC and/or other parts of the breast warrant close examination to rule out malignancy. Biopsy for any persistent lesions is indicated. In case of MPD, further imaging and work-up for search of underlying malignancy is recommended. Management of patients with breast cancer recurrence presenting with MPD is extrapolated from literature of primary and recurrent breast cancers and would typically include either mastectomy or, for select patients with favorable anatomy and time interval, BCS followed by adjuvant re-irradiation with or without endocrine therapy. Expression of ER, PR, and HER2, and further gene expression analysis of both the primary and recurrent tumors are useful in delineating the etiology, although management is similar.
